# Supplementary material for: A generalizable assay for intracellular accumulation to profile cytosolic drug delivery in mammalian cells
Source: Commun Chem. 2026 Feb 6;9:94. doi: 10.1038/s42004-026-01898-8 (PMC12917008; doi:10.1038/s42004-026-01898-8)
Supplement: Supplementary file 3 — Description of Additional Supplementary Files [file 42004_2026_1898_MOESM3_ESM.pdf]

Description of Additional Supplementary Files:

File name: Supplementary Data 1

Description: ChemDraw files for all the chemicals used in this study.

File name: Supplementary Data 2

Description: Raw data for all the figures that were used in the main text.

File name: Supplementary Data 3

Description: Raw data for all the figures that were used in the supplemental text.
